# Green synthesis of ZnS/MoS_2_-decorated porous carbon for photocatalytic degradation of tetracycline under visible light

**DOI:** 10.1038/s41598-025-08920-4

**Published:** 2025-07-01

**Authors:** Zahra Amiri, Hosein Banna Motejadded Emrooz, Mobin Safarzadeh Khosrowshahi

**Affiliations:** Nanotechnology Department, School of Advanced Technologies, Iran University of Science and Technology (IUST), Narmak, Tehran, 16846 Iran

**Keywords:** Tetracycline, MoS_2_, ZnS, Photocatalytic degradation, Porous carbon, Self-activation, Materials science, Materials for energy and catalysis, Nanoscale materials

## Abstract

This study investigated a one-step green pyrolysis method inspired by chemical vapor deposition, utilizing melamine and zinc nitrate precursors with varying sulfur content for degradation and adsorption of tetracycline. The method effectively synthesized a ZnS/MoS_2_ heterojunction composite supported on a porous carbon substrate. It takes advantage of the gases (such as CO_2_, CO, and H_2_O) naturally released during pyrolysis to serve as internal activating agents. In contrast to traditional techniques that rely on inert atmospheres, chemical additives, or complicated high-temperature setups, this method minimizes both the ecological footprint and procedural complexity. The optimal composite, PCS2 (synthesized with 2 g of sulfur), exhibited the lowest band gap of 2.91 eV and the highest specific surface area of 216.83 m^2^ g^−1^, making it the most effective among the tested samples. This composite achieved 55% adsorption of pollutants and demonstrated a total removal efficiency of 81% for tetracycline. The addition of scavengers revealed that the primary active species in the reaction were holes. This synthesized method shows great promise for preparing heterojunction structures, making it highly suitable for removing organic pollutants from contaminated water.

## Introduction

Antibiotics are complex antimicrobial compounds designed to target bacteria or fungi in humans and animals, effectively slowing down or inhibiting the growth of microorganisms^1^. This persistence poses a significant challenge for environmental remediation efforts. Antibiotics can act as a selective force on some microbial populations and affect their resistance (degradability), reproduction, genetics, etc. These substances have been developed to limit the death rate and strengthen the immune systems of humans and animals against the invasion of various bacterial infections. Also, these materials are used in farming to promote growth and control diseases^2,3^. The global daily usage of antibiotics has been on the rise, increasing by 65% between 2000 and 2015, from 21.1 to 34.8 billion doses per day^4^. Antibiotics are seldom fully metabolized by the body, with 30–90% entering the environment as metabolites through urine and feces. Among various antibiotics, tetracyclines (TCs) are one of the most affordable classes, leading to their widespread use, particularly in developing countries^5–7^. TCs are particularly resistant to degradation in soil and water, which raises concerns about their absorption by plants or organisms, allowing them to infiltrate the food chain^8–10^. Additionally, TCs readily bind with ions such as Ca^2+^ to form stable compounds, making their removal more challenging^11^. The presence of tetracycline antibiotics in the environment largely stems from incomplete treatment processes at wastewater facilities^12^.

Given the environmental and public health risks, there is an urgent need for affordable and environmentally friendly solutions to treat water sources contaminated by organic pollutants. This pollution threatens ecosystems and hampers progress towards achieving the United Nations Sustainable Development Goal (UNSDG) of providing clean water and sanitation for everyone^13^. Conventional treatment methods such as adsorption by porous materials, membrane filtration, chemical coagulation, and ion exchange are largely ineffective at completely removing antibiotics^14–16^. These techniques often only transfer pollutants from one phase to another without truly eliminating them, generating unwanted byproducts that require further treatment, thus increasing costs. Even advanced methods like ozonation, while effective, demand high energy consumption^17^. Given these challenges, it is essential to explore alternative technologies for the removal of antibiotics. An ideal strategy should be cost-effective, fast, environmentally friendly, highly efficient at degradation, and scalable for industrial application.

Photocatalytic degradation and adsorption are two of the most efficient processes for removing antimicrobial agents from water sources due to their cost-effectiveness, efficiency, and environmentally friendly characteristics^18–22^. The photocatalytic process involves three key stages: absorption of photons from solar light, generation of electron–hole pairs, and surface catalytic reactions. When a semiconductor is exposed to UV or visible light, it generates electron–hole pairs, producing reactive radicals such as hydroxyl (^·^OH) and superoxide (^·^O_2_^−^). These reactive species can break down harmful contaminants into harmless byproducts like CO_2_ and H_2_O^23–26^. However, this method has some limitations. During the photocatalytic process, nanometer-sized semiconductor particles tend to agglomerate due to their high surface area and surface energy, forming clumps. This reduces the effective surface area and lowers the overall efficiency of the process^27,28^. Moreover, many semiconductor materials exhibit high electron–hole recombination rates, leading to reduced disinfection efficiency. Adsorption, another common treatment method, also has drawbacks, such as pore blockage caused by high pollutant concentrations and the need for costly and time-consuming adsorbent recovery processes^29–33^.

Composite materials that combine semiconductor photocatalysts with high-surface-area adsorbents can partially address the limitations associated with standalone photocatalysts and adsorbents. By leveraging the primary functionalities of both components, these hybrid compounds enhance the removal of contaminants. Additionally, these composites facilitate easier separation of the active materials after treatment, thereby reducing the amount of catalyst required for the process^34,35^. Many factors affect the semiconductors’ photocatalytic degradation efficiency, the main of which are suitable band gaps, i.e., appropriately excitable in the visible range of sunlight irradiation, low level of charge carriers (electron–hole) recombination, and high specific surface area^36,37^. The use of large band gap semiconductors necessitates the use of ultraviolet (UV) light, which is expensive, rare in sunlight, and also harmful to human beings. Therefore, it is preferred that electron–hole pair production be done under visible light or sunlight, which are both free and harmless for humans^38^. The band gap and the electron–hole recombination efficiency of a semiconductor directly depend on the chemical components and, to some extent, on morphology. The effective surface area provides the reaction interface between the charge carriers and the pollutant; therefore, a more effective surface area results in more degradation efficiency^39^. To reduce the energy band gap and electron–hole recombination rate of a photocatalyst, choosing semiconductors with the above-mentioned properties is the first strategy, but this limits the choices for material selection. Engineering the behavior of an existing semiconductor is another solution for this purpose, which seems more effective. Doping semiconductors with appropriate elements, supporting them on suitable scaffolds, and establishing effective connections between multiple semiconductors, such as heterojunctions, core–shell structures, or layered arrangements with several atomic layers, are key strategies in this area^40^.

Hasandoost et al.^41^ synthesized Fe_3_O_4_ modified by (Ce^3+^/Ce^4+^) and then hybridized with graphene oxide (GO) sheets for removing oxytetracycline (OTC) under visible light. The results showed that by adding GO to the composite, degradation increased from 42 to 88%. Also, the specific surface area increased from 41.5 to 55.6 m^2^ g^−1^. GO worked as a support and let the composite have a good distribution, hindering it from agglomeration. With fair conductivity, GO can be considered an electron sink that avoids the recombination of generated electrons and holes. Also, a porous GO scaffold with a large specific surface area provides active sites for the degradation of the pollutant trapped inside the pores. In another experiment, Jiang et al.^42^ prepared 2% carbon nanospheres (CSs)- Bi_2_WO_6_ composite. It was found that by adding CSs to Bi_2_WO_6_, the specific surface area of the product increased from 20.76 to 84.88 m^2^ g^−1^. This composite enhanced the degradation of TC by 25% over pure Bi_2_WO_6_ and reached 84%.

MoS_2_ has attracted considerable interest as a photocatalyst due to its tunable band gap (ranging from 1.1 to 1.9 eV), strong oxidative properties, cost-effectiveness, non-toxicity, and thermal stability. Its unique layered structure, made up of S-Mo-S units, is held together by weak Van der Waals forces, like graphite, enabling efficient photocatalytic activity under visible light. Several studies have shown that reducing the number of MoS_2_ layers increases photocatalytic hydrogen production by exposing more sulfur atoms, improving carrier separation, and boosting electron density in the conduction band. However, the potential of MoS_2_ as a photocatalyst has been limited in industrial settings due to the rapid recombination of photo-induced electrons and holes. Despite this, the material’s large surface area and abundant active sites provide ample opportunities for effective reactions, which are crucial for photocatalysis^43–46^. To overcome the recombination challenge and improve photocatalytic efficiency, MoS_2_ can be combined with another semiconductor to create a heterostructure. This combination enhances solar spectrum absorption and improves the separation of electron–hole pairs. When paired with metal sulfides like ZnS, MoS_2_ demonstrates enhanced photocatalytic performance due to the complementary electronic and catalytic properties of the sulfides. ZnS, with its diverse applications in sensors, photovoltaic cells, biodevices, and photocatalysts, complements MoS_2_ perfectly. Its more negative excited electron potential, efficient generation of electron–hole pairs upon photo-excitation, and biocompatibility make it an excellent partner for MoS_2_. The well-matched lattice parameters of ZnS and MoS_2_ facilitate the formation of intimate interfaces, further boosting the composite’s photocatalytic efficiency^47^. Porous carbon plays a crucial dual role in ZnS/MoS_2_-based photocatalysts. Not only does it act as an excellent electron-conducting support that enhances charge separation and transfer, but it also serves as a highly effective adsorptive substrate. Its large surface area and abundant surface functionalities enable it to adsorb and concentrate organic pollutants near the active sites of the photocatalyst. This localized enrichment promotes faster and more efficient photocatalytic degradation by increasing the contact between the contaminants and the reactive species generated by the ZnS/MoS_2_ heterojunction^37^.

Although various methods have been developed to fabricate different types of photocatalysts and adsorbents, whether individually or in composite form, these approaches often lack environmental sustainability. The synthesis conditions are complex and multi-step, making them less favorable. Furthermore, many of these methods are not suitable for industrial-scale applications, as they remain confined to laboratory settings. This study introduces a green pyrolysis method inspired by chemical vapor deposition (CVD) for the one-step synthesis of a ZnS-MoS_2_ heterojunction photocatalyst supported on hierarchical porous carbon. The process was initiated by preparing a melamine–formaldehyde resin, incorporating zinc nitrate and ammonium heptamolybdate as precursors for the porous carbon-ZnS-MoS_2_ composite. To investigate the structural and morphological features of the synthesized nanocomposite, X-ray diffraction (XRD), Raman spectroscopy, Fourier-transform infrared spectroscopy (FTIR), high-resolution transmission electron microscopy (HRTEM), field emission scanning electron microscopy (FESEM), and nitrogen adsorption–desorption analysis were employed. Additionally, diffuse reflectance spectroscopy (DRS), photoluminescence (PL) spectroscopy, Mott-Schottky analysis, and UV–Visible spectroscopy were used to evaluate the optical and electronic properties of the material. The possible degradation mechanisms were investigated through radical scavenging experiments, identifying the primary active species involved. The synthesized photocatalyst exhibited remarkable adsorption-photocatalytic synergy, achieving 81% removal of tetracycline (TC) within 120 min under visible light.

## Materials and methods

### Materials

Melamine (C_3_H_6_N_6_, 99%), ammonium hepta molybdate tetrahydrate ((NH_4_)_6_Mo_7_O_24_·4H_2_O, 99%), and Sulfur were all obtained from Merck Company (Germany). Zinc nitrate hexahydrate (Zn (No_3_)_2_·6H_2_O, 98%) was purchased from Daejung chemicals Co. Ltd (South Korea). Also, formaldehyde solution (CH_2_O, 37%) was provided by the DR. Mojallali Co. (Iran), and Tetracycline (C_22_H_24_N_2_O_8_) was purchased from Irandaru Co. (Iran).

### Synthesis

#### Synthesis of precursor

The composite precursor was synthesized by the co-precipitation method. In short, 10g of zinc nitrate hexahydrate and 1g of ammonium heptamolybdate tetrahydrate were dissolved in 30 mL of distilled water. Zn (No_3_)_2_·6H_2_O and (NH_4_)_6_Mo_7_O_24_·4H_2_O were Zn and Mo sources, respectively. As the precursor of heteroatoms doped porous carbon, 10g of Melamine (C_3_H_6_N_6_) was dissolved in a mixture of 70 mL of distilled water and 40 mL of formaldehyde solution (CH_2_O), stirred at about 80 °C since fully dissolved. Then, the first solution was added, and stirring continued until the formation of a white precipitate. Finally, the white powder was put in an oven for 8 h at 80 °C.

#### Synthesis of C/ZnS/MoS_2_ nanocomposite

**Fig. 1:**
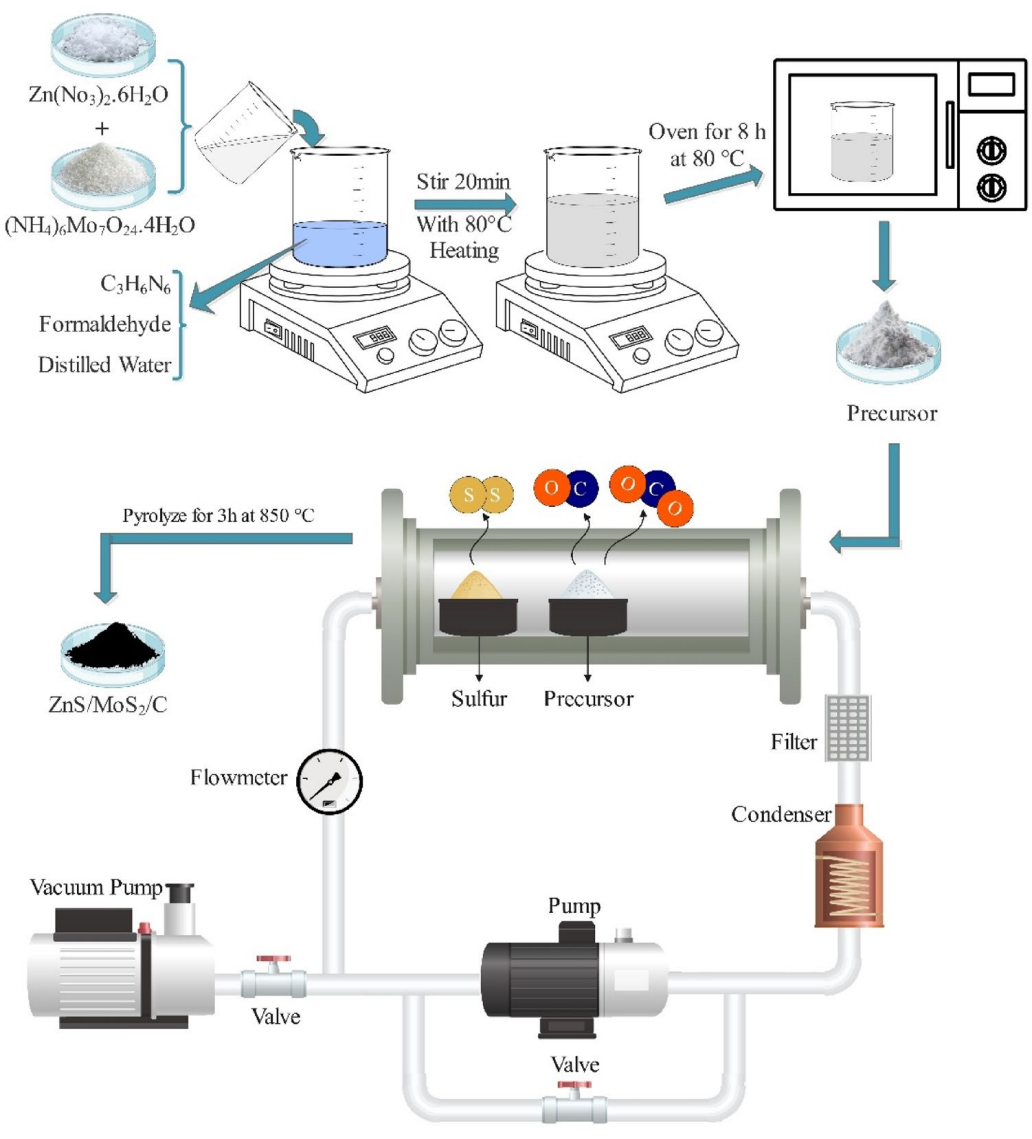
A schematic illustration of the nanocomposite synthesis process using a self-activation furnace. The green pyrolysis system contains a vacuum pump, condenser, tube electrical furnace, flowmeter, and filter.

The ZnS-MoS_2_ nanocomposite supported on porous carbon was synthesized using a one-step self-activation/chemical vapor deposition (CVD) method. This procedure was executed within a specially designed self-activating synthesis system, as illustrated in Fig. 1. The synthesis setup included an electrical tube furnace, a vacuum pump for evacuating the initial atmosphere, an air pump for circulating the furnace atmosphere during heating, a condenser, and various filters to capture pollutants and regulate system pressure. The self-activation method offers a simple, eco-friendly alternative for producing porous carbon materials by utilizing gases (CO_2_, CO, H_2_O) released during pyrolysis as internal activating agents. Unlike conventional methods that require inert gases, chemical agents, or complex high-temperature equipment, self-activation reduces environmental impact and operational complexity. The porous structure formed through this process provides an ideal substrate for the efficient loading and uniform dispersion of functional components, such as metal sulfides or oxides, thereby facilitating the one-step fabrication of nanocomposites. A total of 10 g of the previously prepared sediment was pyrolyzed for 3 h at 850 °C in the furnace’s hot zone. Concurrently, specific amounts of elemental sulfur (0, 2, and 5 g) were placed in designated areas of the furnace at approximately 250 °C. Following the pyrolysis process, the furnace was allowed to return to ambient conditions. The composites, with varying sulfur content, were labeled as PCS0, PCS2, and PCS5.

### Photocatalytic experiments

The photocatalytic degradation of TC by the PCS2 composite was evaluated under visible light irradiation using three 30W LED lamps (λ > 410 nm) as the light source at room temperature. The distance between the sample and the light source was approximately 10 cm. Notably, no cut-off filter was employed to block ultraviolet light. A 10 mg sample of the composite powder was dispersed in 100 mL of a TC solution (20 mg L^−1^), followed by stirring in the dark for 30 min to establish adsorption–desorption equilibrium. Afterward, the suspension was exposed to light, and 5 mL samples were collected at various time intervals (0, 15, 30, 45, and 60 min) for further analysis. The filtrates were examined at the absorption wavelength of 357 nm using a UV–vis spectrophotometer (UV–Vis DRS, S-4100, SCINCO, South Korea). To investigate the degradation mechanism and identify the most active radicals involved, different scavengers were employed. Ethanol and Na_2_SO_4_ were used as scavengers for holes (h⁺) and electrons (e⁻), respectively. The degradation efficiency can be calculated as follows Eq. (1):

1 $$Degradation Efficiency (\%)=\left(1-\frac{{C}_{t}}{{C}_{0}}\right)\times 100\%$$

### Degradation kinetics

The kinetics for the photocatalytic degradation of TC by PCS2 composite under light irradiation was investigated, and the obtained data were fitted by a pseudo-second-order kinetic equation as follows Eq. (2):

2 $$\frac{1}{{C}_{t}}=\frac{1}{{C}_{0}}+Kt$$

where C_0_ (mg L^−1^) is the initial TC concentration before irradiation, C (mg L^−1^) is the TC concentration at the time, t (min) under light irradiation, and k (min^−1^) is the pseudo-second-order rate constant.

### Characterization

For the examination of the material phase and crystallinity, the material was analyzed by X-ray diffraction (XRD) patterns using D8 Advance, Bruker German, with LYNXEYE-XE-T detector and 0.0001 steps. N_2_ adsorption/desorption (Brunauer Emmett Teller, Belsorp mini II, Microtrac Bel Corp, Japan) was used for determining the volume of specific surfaces and the surface area of meso and micropores. FTIR (Thermo-Avatar) was employed to determine the chemical bonding and verify the presence of elements and complexes. Ultraviolet–visible diffuse reflectance spectroscopy (UV–Vis DRS, S-4100, SCINCO, South Korea) was used to estimate light absorption and to determine its band gap by the Tauc equation. Ultraviolet–visible spectroscopy is used to determine the removal of antibiotics from the solution. RAMAN (Technooran, Microspectrophotometer-ram) is used to confirm the presence of elements (especially carbon) in the material. Photoluminescence (PL) (Varian PL-XT) has been taken to estimate the recombination of holes and excited electrons and the presence of existing MoS_2_ and ZnS. Field emission scanning electron microscopy (FESEM) (MIRA3, TESCAN) was used to examine the morphology of the composite. The Mott-Schottky plot was used to show the location of the valence and conduction bands and to examine the density of charge carriers of the sample. Structural morphology was examined by High-resolution transmission (HRTEM) (FEI TECNAI F20).

### Electrochemical measurements

All electrochemical characterizations were conducted using a Metrohm DropSens µStat-i 400 s with a three-electrode system. A platinum (Pt) wire served as the counter electrode, while an Ag/AgCl electrode was used as the reference electrode. Mott-Schottky measurements were carried out in a 0.1 M Na_2_SO_4_ electrolyte solution at a frequency of 1000 Hz with an alternating current (AC) voltage amplitude of 5 mV.

## Results and discussion

### Material characterizations

**Fig. 2:**
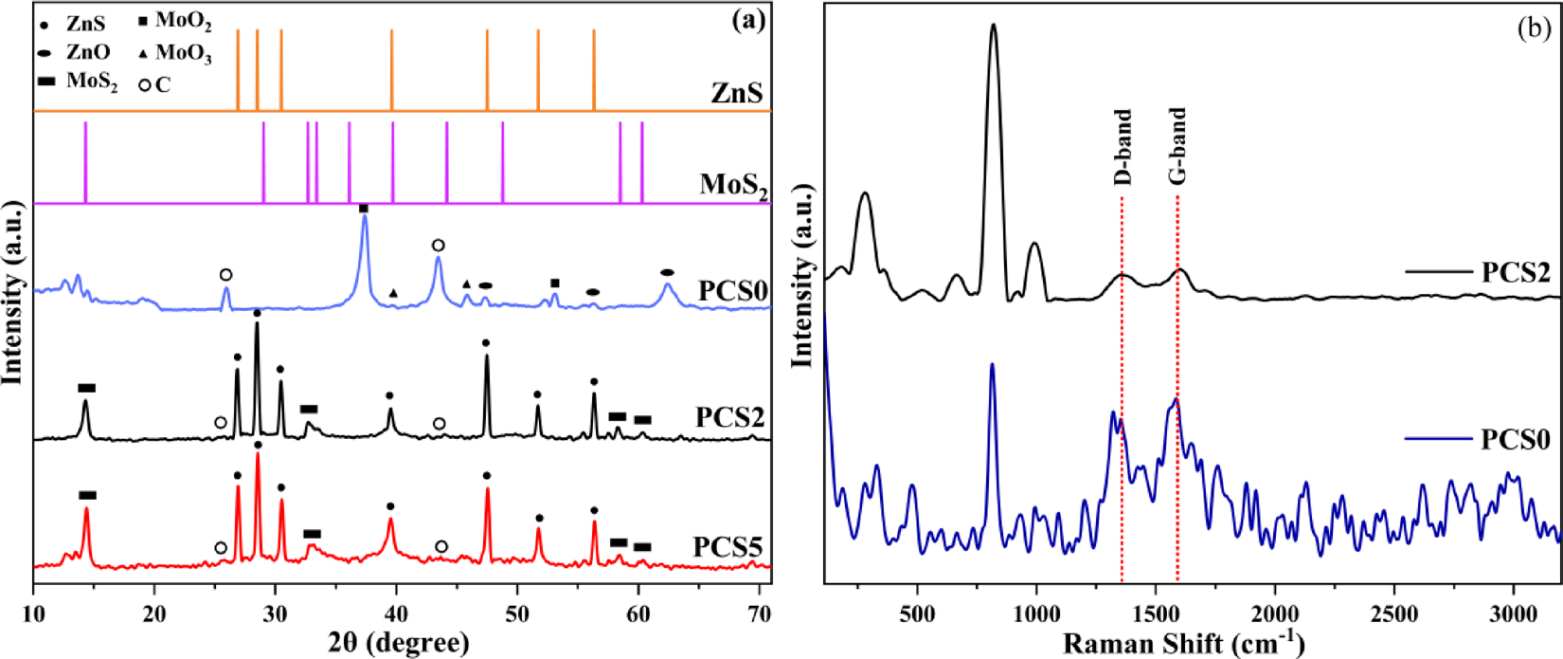
(**a**) XRD patterns of PCS0 (sample without sulfur), PCS2 (synthesized by 2 g sulfur), and PCS5 (synthesized by 5 g sulfur) composites, and (**b**) Raman spectrum of PCS0 and PCS2 composites.

XRD and RAMAN analysis were applied to characterize the crystalline structure of the synthesized compounds, the results of which have been presented in Fig. 2. Also, by analyzing diffraction patterns, present phases in a sample can be identified. In this study, the XRD patterns of three different samples, PCS0, PCS2, and PCS5 composites, were examined and illustrated in Fig. 2a. In the PCS2 and PCS5 composites, distinct peaks corresponding to ZnS and MoS_2_ were observed, while the PCS0 composite exhibited peaks for ZnO, MoO_2_, and MoO_3_, indicating a different composition compared to PCS2 and PCS5. The diffraction peaks at 26.8, 28.4, 30.4, 39.5, 47.4, 51.7, and 56.3° respectively correspond to the (100), (002), (101), (102), (110), (103), and (112) crystal planes of the hexagonal wurtzite ZnS (JCPDS 36-1450)^48^. The peaks of MoS_2_ at 14.28, 32.6, 58.3, and 60.3° correspond to the (002), (100), (110), and (008) planes and can be assigned to the characteristic peaks of 2H-MoS_2_ (JCPDS 37-1492), respectively^49^. In particular, the ordered stacking of S-Mo-S layers is suggested by the peak at 14.2° is indicative of the (002) crystalline plane of MoS_2_^50^. The peaks of 37.03 and 53.01° correspond to the (211) and (312) planes of MoO_2_, (JCPDS 01-078-1071) respectively^51^, and the peaks of 33.76, 39.4, and 45.8° respectively correspond to the (111), (060), and (200) planes of MoO_3_. Moreover, the peaks of 47.3, 56.2, and 62.4° correspond to the (102), (110), and (103) planes of ZnO, (JCPDS 36-1451), respectively^52,53^. The diffraction peaks at 26.5 and 44.3° can be indexed to the (100) and (002) planes of carbon (JCPDS75-1621)^54^. After synthesizing the PCS0 composite, because of the difference between the ionic diameters of materials, the location of the carbon peaks has been shifted to a higher 2θ angle^55^. In the XRD pattern of the PCS2 nanocomposite, a good coexistence of two phases (ZnS and MoS_2_) has been shown, indicating that S was successfully introduced into the system. In addition, most of the MoS_2_ diffraction peaks and some of the ZnS diffraction peaks’ intensities increased with increasing weight ratios of the S element. Moreover, the addition of more sulfur increased crystallite sizes. Based on the Debye–Scherrer equation, the growth was more prominent for MoS_2_, with its crystallite size increasing significantly from 11.6 to 15.7 nm. In contrast, the crystallite size of ZnS showed only a slight increase, from 24.3 to 25.5 nm.

Raman spectroscopy was employed to further analyze the structure of the synthesized particles, particularly the degree of graphitization in the carbon matrix, which was not discernible in the XRD analysis. The carbon framework is critical as an electron conductor in the composite. Figure 2b presents the Raman spectra for the PCS0 and PCS2 composites. Two characteristic bands, observed at approximately 1350 cm^−1^ and 1590 cm^−1^, correspond to the D-band, associated with sp^3^-hybridized carbon, and the G-band, related to the in-plane vibrations of sp^2^ carbon atoms^56^. Notably, the intensity ratio of the D band to the G band (I_D_/I_G_) is used to assess the graphitization degree of the carbon material. For the PCS0 and PCS2 composites, the I_D_/I_G_ ratio is 0.85 and 0.84, respectively. The PCS0 sample exhibits a higher I_D_/I_G_, indicating lower graphitization and increased structural defects^57^. In addition to the carbon-related peaks, Raman spectroscopy revealed the presence of other compounds that were not detected in the XRD analysis. Specifically, the stretching vibration modes of MoO_2_ were observed at 663, 815, and 992 cm^−1^. Additionally, characteristic finger bands at 281, 331, and 477 cm^−1^ correspond to the phonon vibration modes of MoO_2_. These findings highlight the ability of Raman spectroscopy to identify phases not visible in XRD, providing a more comprehensive understanding of the composite’s structure^58^. Finally, the distinct peak at 360 cm^−1^ corresponds to the E_12_g vibration mode, representing the in-plane displacement of Mo and S atoms. The reduction in Raman peak intensity observed in PCS2 compared to PCS0 (the sulfur-free sample) can be explained by the formation of sulfur-related defects. These imperfections locally disrupt the ZnS lattice, resulting in a weaker Raman signal as fewer phonons can coherently contribute to the scattering process. Moreover, the presence of sulfur vacancies is likely to introduce additional non-radiative recombination centers, further diminishing the Rama intensity^59^.

**Fig. 3:**
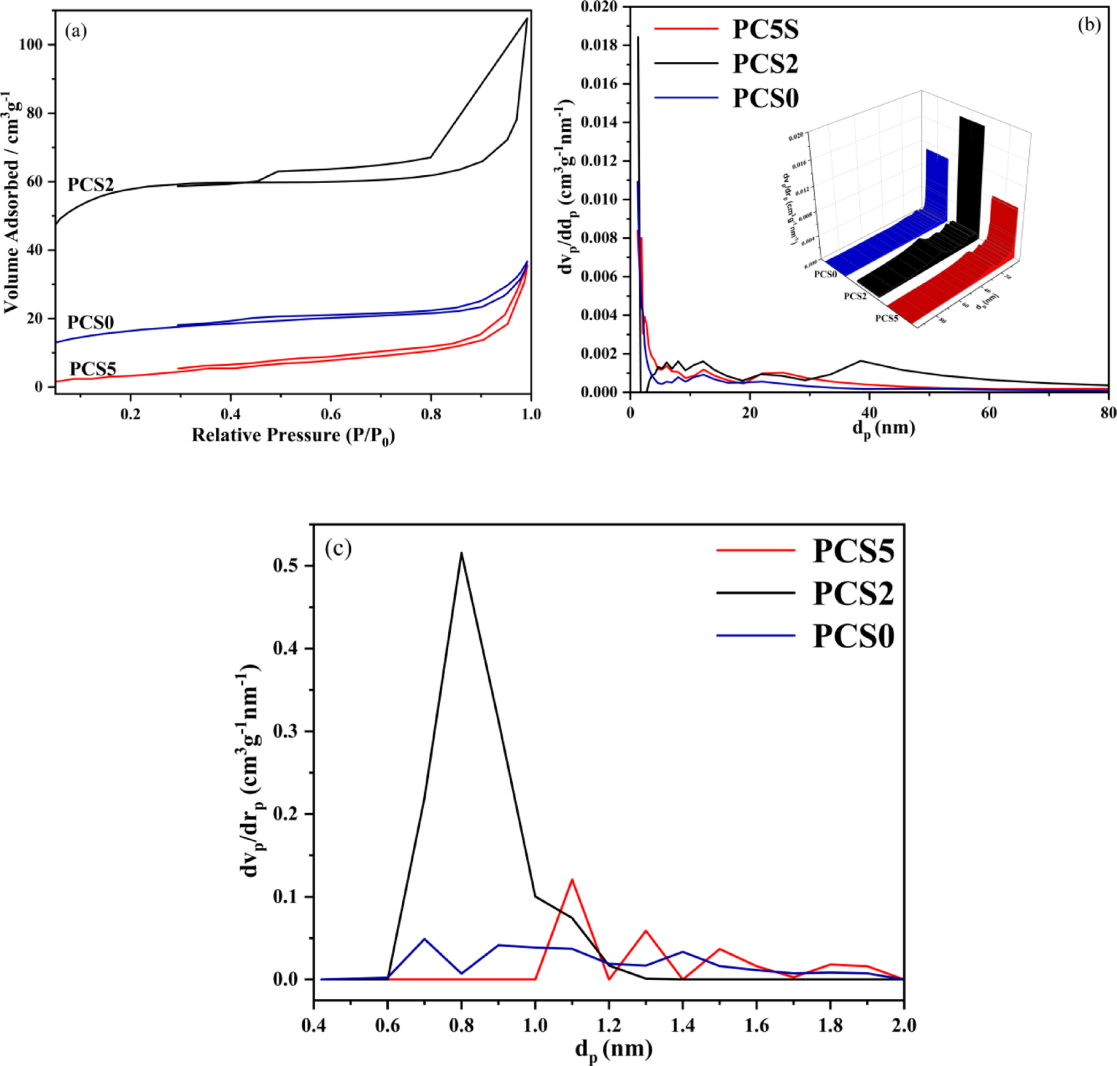
(**a**) N_2_ adsorption–desorption isotherm, (**b**) BJH diagram for assessing pore size distribution, and (**c**) MP plot (Micropore Plot) of PCS0, PCS2, and PCS5 composites.

The porous structure of the synthesized composites has been evaluated using N_2_ adsorption–desorption isotherm analysis. Figure 3a–c display the N_2_ adsorption–desorption isotherm, BJH pore size distribution, and micropore plot (MP). Also, the specific surface area and porosity parameters are listed in Table 1**.** The dramatic uptake of N_2_ at a low relative pressure (P/P_0_ < 0.02) represents the typical characteristic of micropores, and the continuous uptake of N_2_ at P/P_0_ > 0.05 reveals the N_2_ adsorption in mesopores. Among the samples, PCS2 displays a more pronounced hysteresis loop at P/P₀ > 0.40^60^, indicating the presence of more significant mesopores, as shown in Fig. 3a. This composite’s high specific surface area and interconnected porous structure contribute to enhanced photocatalytic activity and improved efficiency in pollutant degradation^61^. These characteristics make PCS2 particularly effective for environmental applications. PCS0, PCS2, and PCS5 composites have a specific surface area of 58.69, 216.83, and 17.06 m^2^g^−1^, the volume of mesoporosity and microporosity for the PCS0 sample are, 0.03 cm^3^ g^−1^ and 0.02 cm^3^ g^−1^, for the PCS2 sample, 0.124 cm^3^ g^−1^ and 0.036 cm^3^ g^−1^, and for the PCS5 sample, 0.027 cm^3^ g^−1^ and 0.023 cm^3^ g^−1^. The PCS2 composite shows both small and large pores. These results indicate a more pronounced hierarchical porous structure of the PCS2 composite. A hierarchical porous structure can reduce tortuosity and provide appropriate channels for wastewater penetration. The micropore volume drastically increases with the increase of sulfur addition. Generally, the addition of sulfur induces an effective etching effect on carbon precursors during carbonization. By increasing the sulfur ratio, the pore diameter increased. This can confirm the etching process. The result revealed that the appropriate addition of sulfur could increase the specific surface area of the sample from 58.69 to 216.83 m^2^ g^−1^, but it has an optimum amount, and beyond an optimum sulfur amount, the surface area decreases. There are two possibilities for that: (I) By reacting with oxygen, sulfur turns into SO_2_, which causes oxygen to play a limiting role and prevent the formation of CO_2_, which is one of the main factors for porosity formation. (II) The other possible reason for the observed trend in the dependency between the specific surface area and the sulfur content is that sulfur acts as an etching factor, and with excessive etching, the surface is destroyed, and as a result, the specific surface area is reduced. Because the PCS2 composite has a higher specific surface area, it is expected to provide more active sites for photocatalytic reaction and have a higher photocatalytic efficiency.

**Table 1:** Textural properties of PCS0, PCS2, and PCS5 composites.

Samples	S_BET_ (m^2^ g^−1^)	S_mic_ (m^2^ g^−1^)	S_mes_ (m^2^ g^−1^)	V_total_ (cm^3^ g^−1^)	V_mic_ (cm^3^ g^−1^)	V_mes/mic_ (cm^3^ g^−1^)	D_pore_ (nm)
PCS0	58.69	19.23	32.92	0.05	0.02	1.5	3.73
PCS2	216.83	42.42	162.59	0.16	0.036	3.33	2.99
PCS5	17.06	5.66	2.77	0.05	0.023	1.17	12.15

**Fig. 4:**
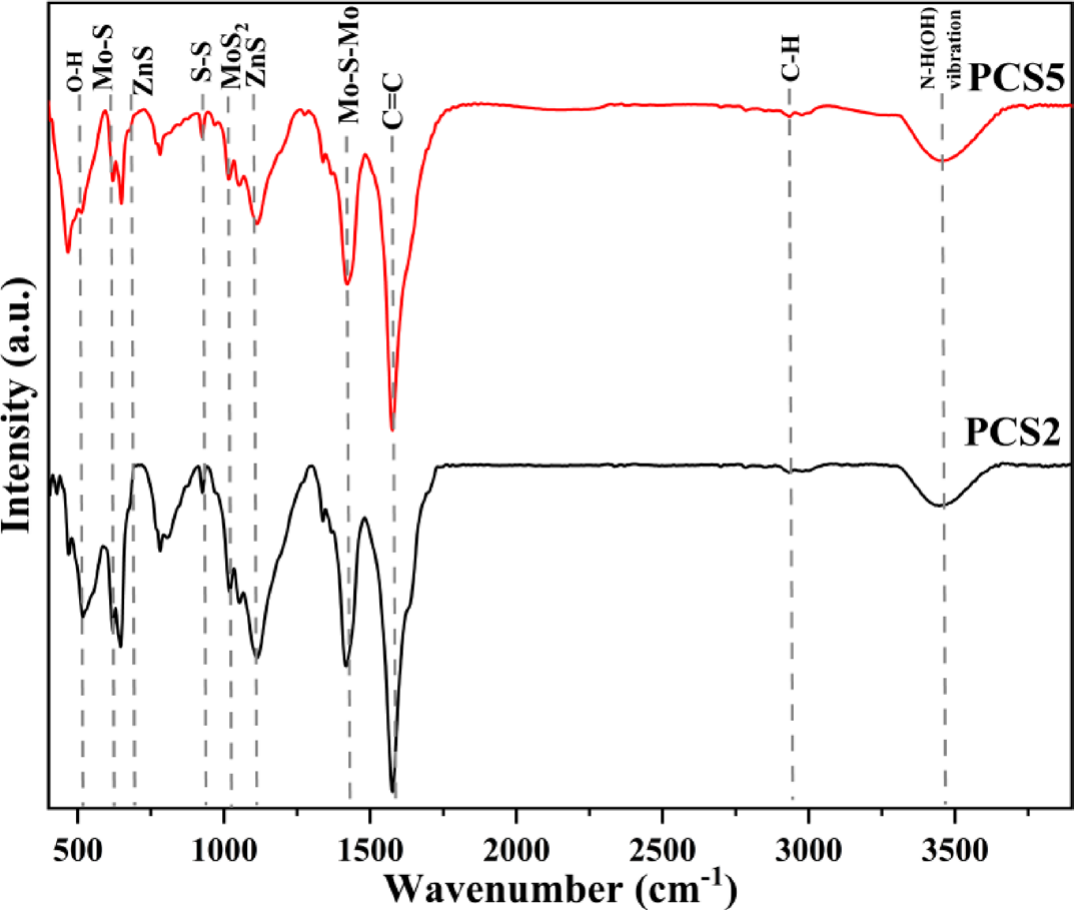
FTIR spectra of PCS2 and PCS5 composites.

The surface functional groups of the PCS2 and PCS5 composites collected from FTIR spectroscopy are shown in Fig. 4. The peak at ~ 3420 cm^−1^ is consistent with the O–H stretching vibrations of hydroxyl groups^62^. Furthermore, N–H stretching vibrations at 3230 cm^−1^ overlap with O–H bands (51). Also, peak 2940 cm^−1^ corresponds to the C-H vibrational stretching bond^63^. The peak centered at 553 cm^−1^ is attributed to the in-plane bending vibrations of the O–H group^64^. The peak at 1110 cm^−1^ can be related to the C–O vibration stretching bond and can also indicate the presence of ZnS in the structure. The peak observed in 1577 cm^−1^ corresponds to the C=C vibration stretching bond in the carbon ring structure, and the peak in 1640 cm^−1^ shows the stretching vibrations of the C=C ring. The 650 and 1015 cm^−1^ peaks are related to sulfides and indicate the Zn–S bond. Due to the high temperature of the system, most of the functional groups have decomposed and formed CO and CO_2_ volatile gases. The 1415 and 650 cm^−1^ peaks are related to Mo-S and Mo-S-Mo, respectively, confirming the formation of MoS_2_^63^.

**Fig. 5:**
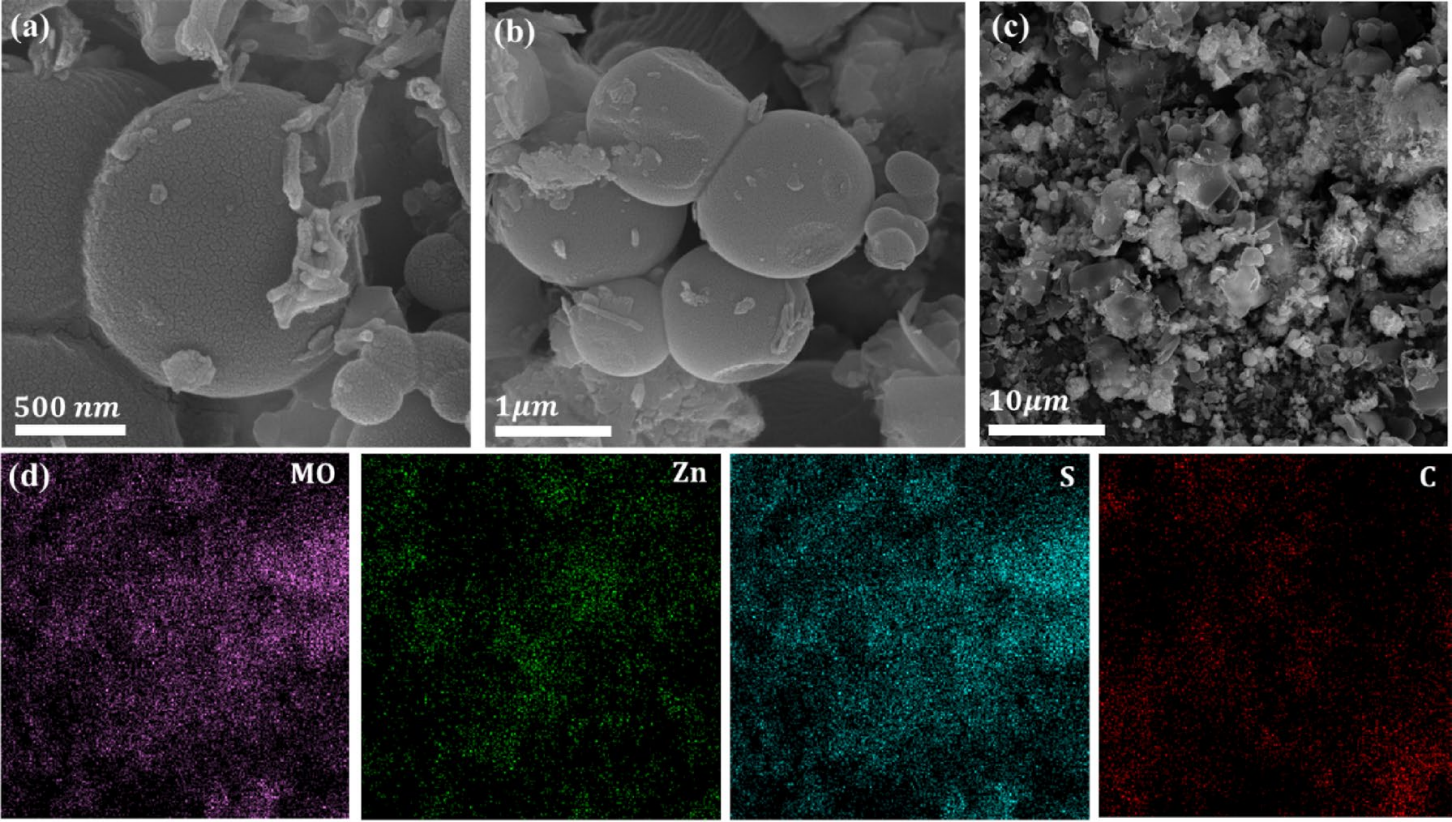
(**a**–**c**) FE-SEM micrographs of PCS2 composite, and (**d**) elemental mapping of Mo, Zn, S, and C elements, respectively.

The morphological structure of the PCS2 composite, examined via FESEM (shown in Fig. 5a–c), reveals a hierarchical porous architecture that aligns with the BJH plot of the composite. Spherical nanoparticles, likely associated with ZnS, along with carbon sheets, were observed. Elemental mapping (Fig. 5d) from Fig. 5c confirms the successful positioning of ZnS and MoS₂ nanoparticles on the carbon sheets, illustrating their distribution within the carbon framework. This suggests that ZnS and MoS_2_ are well-integrated and likely to form a heterojunction nanocomposite structure. Also, MoS_2_ exhibits a layered, sheet-like morphology due to its inherent 2D hexagonal crystal structure. These layers form a morphology that resembles thin, flaky sheets or stacked platelets (Fig. 5b)^65^. The interconnected framework within the porous carbon can improve visible light absorption and provide more active sites. At the same time, the intimate interface between the carbon matrix and ZnS particles facilitates efficient electron transfer and supports the effective separation of photo-generated charge carriers^36^.

**Fig. 6:**
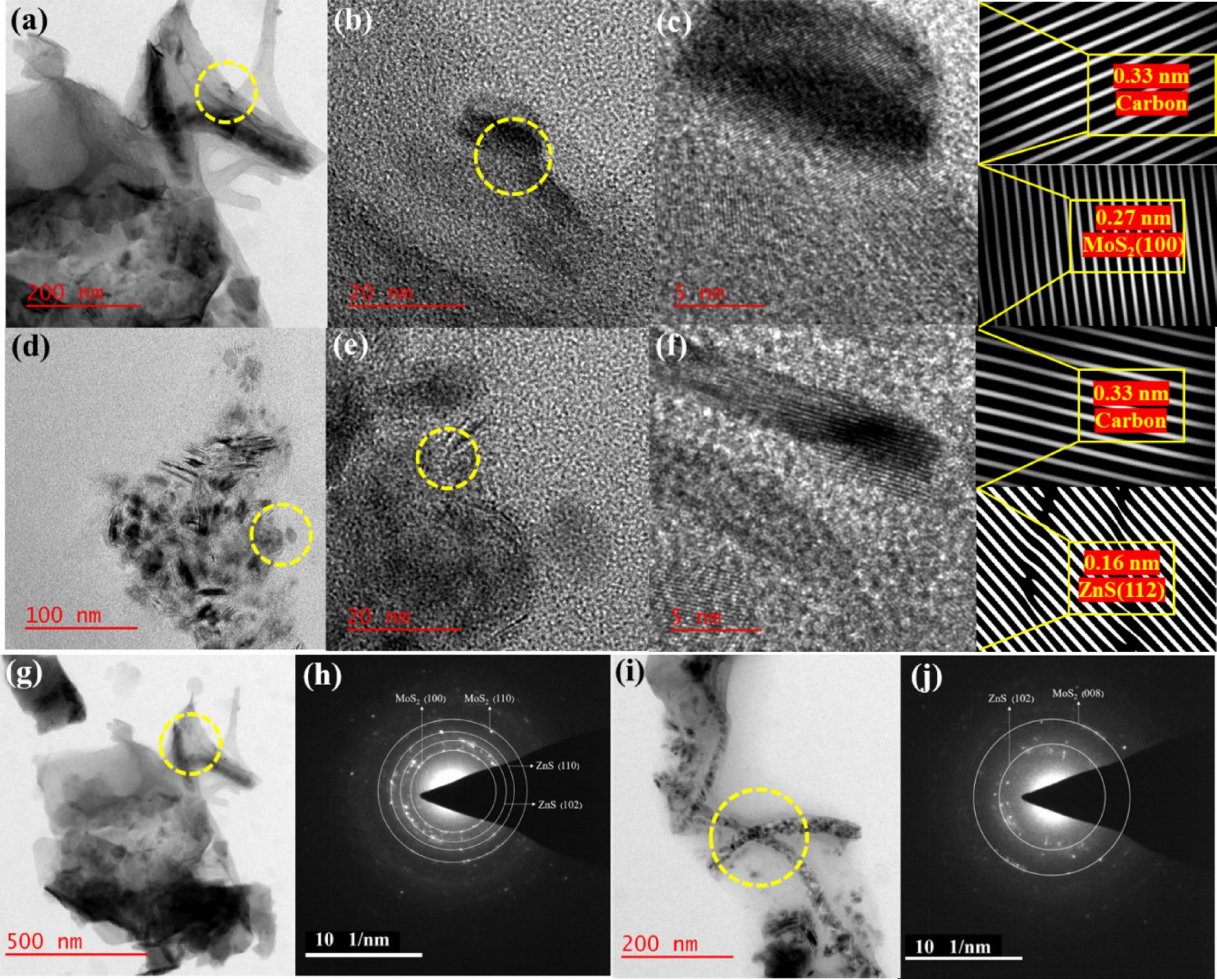
(**a**–**f**, **g**, and **i**) HRTEM images and (**h**, and **j**) SAED (Selected Area Electron Diffraction) of PCS2 composite.

The detailed structural information was also detected by HRTEM analysis. HRTEM of PCS2 composite displays a large-scale sheet-like structure, which can be seen in Fig. 6. Based on the HRTEM micrographs, the lattice spacings of the composite components were determined as follows: porous carbon exhibits a lattice spacing of 0.33 nm, ZnS shows a spacing of 0.16 nm, and MoS_2_ has a spacing of 0.27 nm. These distinct lattice spacings confirm the formation of a heterojunction structure, indicating that ZnS and MoS₂ are well integrated with the carbon matrix. In addition, Fig. 6j shows the selected area electron diffraction (SAED) pattern corresponding to (102) and (008) planes of ZnS and MoS_2_, in which diffraction peaks in the XRD pattern occurred at 39.5° and 60.3°, respectively. These results were proof of the formation of ZnS/MoS_2_ heterojunctions.

A comparison of the d-spacing values obtained from XRD and HRTEM methods was performed. Bragg’s equation ($2d\mathit{sin}\uptheta =n\lambda )$ was applied to estimate the d-spacing from XRD patterns, while ImageJ software was utilized to measure the d-spacing from HRTEM and SAED images. The results from both techniques show good consistency, and the corresponding values are provided in Table 2.

**Table 2:** Comparison of d-Space values from XRD and HRTEM.

(hkl)	2θ (°)	d (XRD) (Å)	d (HRTEM) (Å)
MoS_2_ (100)	32.85	2.7	2.67
ZnS (102)	39.54	2.2	2.24
ZnS (110)	47.55	1.9	1.87
ZnS (103)	51.77	1.7	1.66

### Mechanism of composite synthesis

At the first step, all exhaust paths are closed, and the heating process has begun. By increasing the temperature, the organic elements in the precursor have been evaporated and circulated in the system with the help of a pump. During heat treatment, melamine undergoes sequential transformations, forming melam (350 °C), melem (450 °C), melon (600 °C), and g–C_3_N_4_ (> 600 °C), accompanied by the release of NH_3_. After removing residual salt, activated carbon with a hierarchical pore structure is obtained^57^. Also, some other possible reactions may happen during the heating process^66–68^:

3 $${\text{H}}_{{2}} {\text{O}}_{{({\text{l}})}} \to {\text{H}}_{{2}} {\text{O}}_{{({\text{g}})}}$$

4 $${\text{C}}_{{({\text{s}})}} + {\text{ H}}_{{2}} {\text{O}}_{{({\text{g}})}} \to {\text{CO}}_{{({\text{g}})}} + {\text{ H}}_{{{2}({\text{g}})}}$$

5 $${\text{2C}}_{{({\text{s}})}} + {\text{ O}}_{{{2}({\text{g}})}} \to {\text{2CO}}_{{({\text{g}})}}$$

6 $${\text{2CO}}_{{({\text{s}})}} + {\text{ O}}_{{{2}({\text{g}})}} \to {\text{2CO}}_{{{2}({\text{g}})}}$$

7 $${\text{CO}}_{{({\text{g}})}} + {\text{ H}}_{{2}} {\text{O}}_{{({\text{g}})}} \to {\text{CO}}_{{{2}({\text{g}})}} + {\text{ H}}_{{{2}({\text{g}})}}$$

8 $${\text{C}}_{{({\text{s}})}} + {\text{ CO}}_{{{2}({\text{g}})}} \to {\text{2CO}}_{{({\text{g}})}}$$

MoS_2_ can form through two primary pathways: in the first, MoO_3_ is reduced to MoO_2_, which reacts with sulfur vapor to produce MoS₂. In the second pathway, MoO_2_ directly undergoes sulfurization to form MoS₂. Here, MoO_2_ plays a key role as an intermediate, facilitating the surface formation of MoS_2_. Notably, at 650 °C, the Gibbs free energy of reaction (9) is more negative than that of reaction (10), suggesting it is the more thermodynamically favorable pathway under these conditions^69^.

9 $${\text{4MoO}}_{{{3}({\text{v}})}} + {\text{7S}}_{{{2}({\text{v}})}} \to {\text{ 4MoS}}_{{{2}({\text{s}})}} + {\text{6SO}}_{{{2}({\text{v}})}}$$

10 $${\text{4MoO}}_{{{2}({\text{v}})}} + {\text{6S}}_{{{2}({\text{v}})}} \to {\text{ 4MoS}}_{{{2}({\text{s}})}} + {\text{4SO}}_{{{2}({\text{v}})}}$$

The formation of ZnS happens through the thermal treatment of ZnO. In the first step, ZnO reacted with the initially produced CO and formed Zn^68^. Wurtzite ZnS is created when Zn combines with in-situ reactive sulfur and oxygenated species that are released during the thermal decomposition of both zinc and molybdenum salt. At 850 °C, reaction (6) has a more negative Gibbs energy than reaction (12) ^66^. It means the first reaction has priority, and the available oxygen is used to react with the organic substances. As a result, all the Zn is involved in the reaction with sulfur. Possible reactions to form ZnS^66,68^:

11 $${\text{ZnO}}_{{({\text{s}})}} + {\text{ CO}}_{{({\text{g}})}} \to {\text{ Zn}}_{{({\text{g}})}} + {\text{ CO}}_{{{2}({\text{g}})}}$$

12 $${\text{2 Zn}}_{{({\text{g}})}} + {\text{ O}}_{{{2}({\text{g}})}} \to {\text{ 2 ZnO}}_{{({\text{s}})}}$$

13 $${\text{Zn}}_{{({\text{g}})}} + {\text{ S}}_{{{2}({\text{g}})}} \to {\text{ ZnS}}_{{({\text{s}})}}$$

14 $${\text{ZnO}}_{{({\text{s}})}} + {\text{S}}_{{{2}({\text{g}})}} \to {\text{ ZnS}}_{{({\text{s}})}} + {\text{ O}}_{{{2}({\text{g}})}}$$

15 $${\text{ZnO}}_{{({\text{s}})}} + {\text{SO}}_{{{2}({\text{g}})}} + {\text{C}}_{{({\text{s}})}} \to {\text{ ZnS}}_{{({\text{s}})}} + {\text{ CO}}_{{{2}({\text{g}})}}$$

Thermally activated porous carbon sheets were used as support for nucleation sites of ZnS/MoS_2_, leading to a homogeneous dispersion of nanoscale heterostructured particles onto carbon, as can be seen in elemental mapping.

### Optical and photo-electrochemical properties

**Fig. 7:**
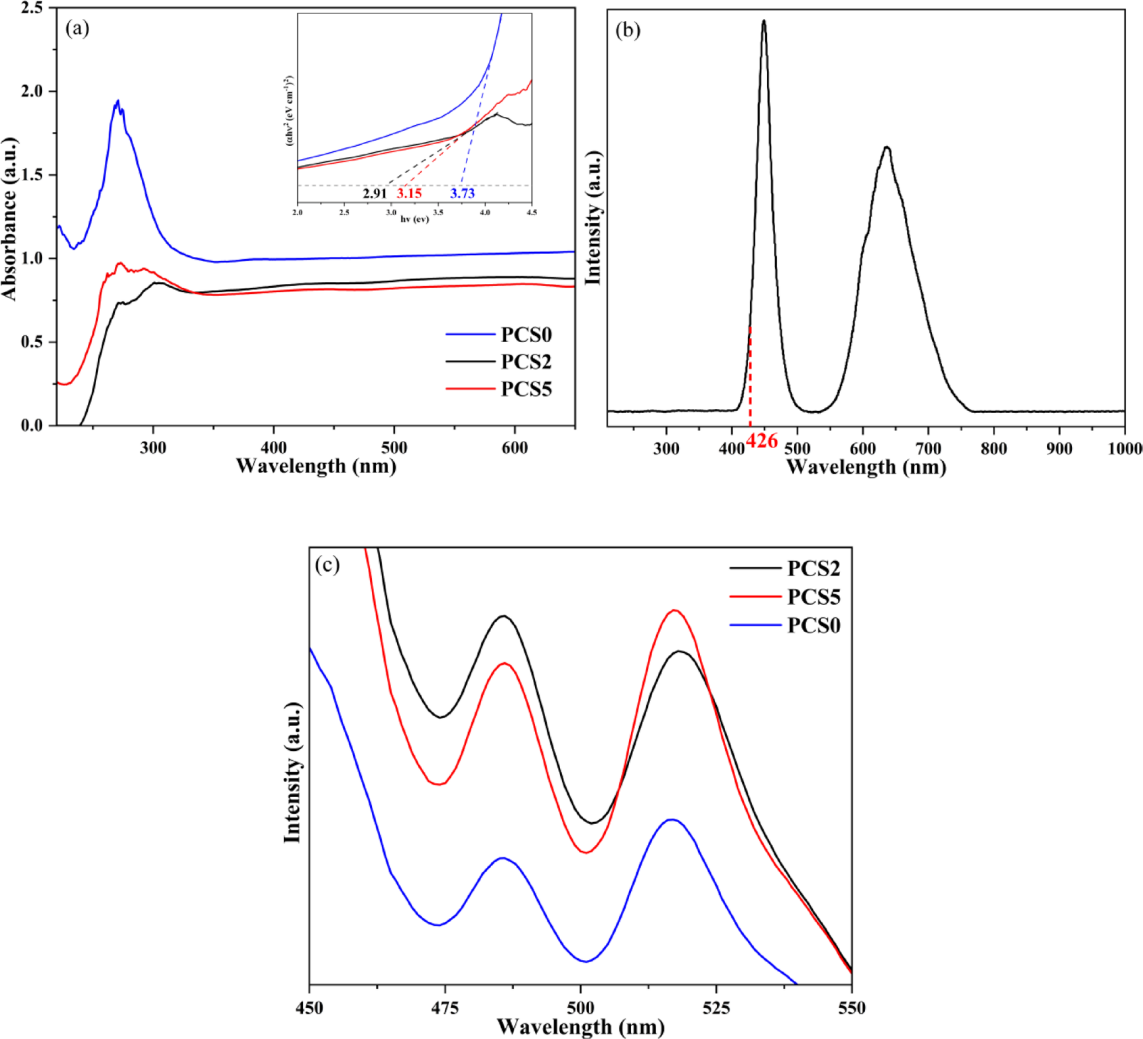
(**a**) UV–Vis Diffuse Reflectance Spectroscopy with inserted band gap determination plotting by the Tauc equation for PCS0, PCS2, PCS5 composites, (**b**) spectra of the lamp, and (**c**) photoluminescence spectra of PCS0, PCS2, PCS5 composites.

The optical properties of the samples were characterized using UV–Vis diffuse reflectance spectroscopy (UV–Vis DRS), as shown in Fig. 7a. Composites exhibit very similar absorption spectra except for the composites with PCS0. The absorption edges of the PCS5, PCS0, and PCS2 tests were 570, 352, and 525 nm, respectively. The bandgap edge (E_g_) of a semiconductor can evaluate the electron–hole pairs’ production and transformation. The relationship between band edge and light absorption (The Tauc plot) could be described by the following formula Equation^70^:

16 $$\alpha hv=K{\left(hv-{E}_{g}\right)}^{n/2}$$

where α, h, υ, K, n, and Eg are the absorption coefficient, Planck constant, light frequency, constant, electronic transition, and the band gap, respectively. Considering that these composites are typical indirect band gap semiconductors, the n value for the indirect bandgap is 2, and the value for this experiment is 0.5, which is related to the direct bandgap. The changes of (αhυ)^2^ as a function of the energy of incident photons (hυ) were depicted in Fig. 7a, and the E_g_ values of PCS5, PCS0, and PCS2 were 3.15, 3.73, and 2.91 eV, respectively. After the modification of composites by adding sulfur, band gaps became narrower, which is more suitable for use in visible light. Also, adding an S element causes a reduction of light absorption. Incorporating sulfur atoms into the composite structure lowers the conduction band minimum or raises the valence band maximum, effectively narrowing the band gap and enhancing visible-light absorption. Sulfur also stabilizes defect states within the band gap, particularly at phase interfaces, which act as intermediate energy levels, reducing the energy needed for electron excitation. Additionally, sulfur improves electron mobility and reduces electron–hole recombination, thereby enhancing charge separation and boosting photocatalytic efficiency under visible light^71^. The PCS2 composite stands out as the most effective sample, with a bandgap of 2.91 eV (420 nm), making it highly responsive to visible light (~ 400–750 nm). Figure 7b illustrates the lamp spectrum used, revealing two peaks within the visible range, which align well with the bandgap edge of the PCS2 composite, also marked on the diagram. This alignment supports the composite’s strong visible-light performance.

Photoluminescence (PL) spectroscopy has been widely employed to study the photo-generated charge carriers’ excitation and transfer in photocatalysis over semiconductors. Moreover, PL spectroscopy is utilized to measure the light emitted by the material as excited electrons drop back to the ground state. A higher intensity of PL is associated with a higher rate of radiative recombination. A lower intensity of PL indicates fewer radiatively recombining photogenerated electrons and holes. This means that a higher percentage of these charge carriers are available to participate in the surface reaction of interest, and hence, there will be higher photocatalytic activity. Thus, reduced PL intensity is, in general, a desirable quality for efficient photocatalysts^72,73^. Figure 7c presents the PL spectra of the PCS0, PCS2, and PCS5 nanocomposites with an excitation wavelength of 380 nm. In contrast, nanocomposite PCS0 has the lowest PL peaks. This means the PCS0 nanocomposite has the lowest recombination and better charge transfer efficiency. The PL signals of the two other nanocomposites were approximately the same. The result indicated that the recombination rate of photoinduced electrons and hole pairs was higher when S was added to the nanocomposite. It seems that sulfur atoms can create impurity levels or defect sites within the band structure of the nanocomposite. These newly formed shallow traps serve as intermediate states that facilitate the return of electrons to the valence band, thereby enhancing the recombination process^74^. Despite the lower rate of recombination, the extremely low surface area and wide band gap made it difficult to explore the photocatalytic behavior of the sulfur-free sample under visible light.

**Fig. 8:**
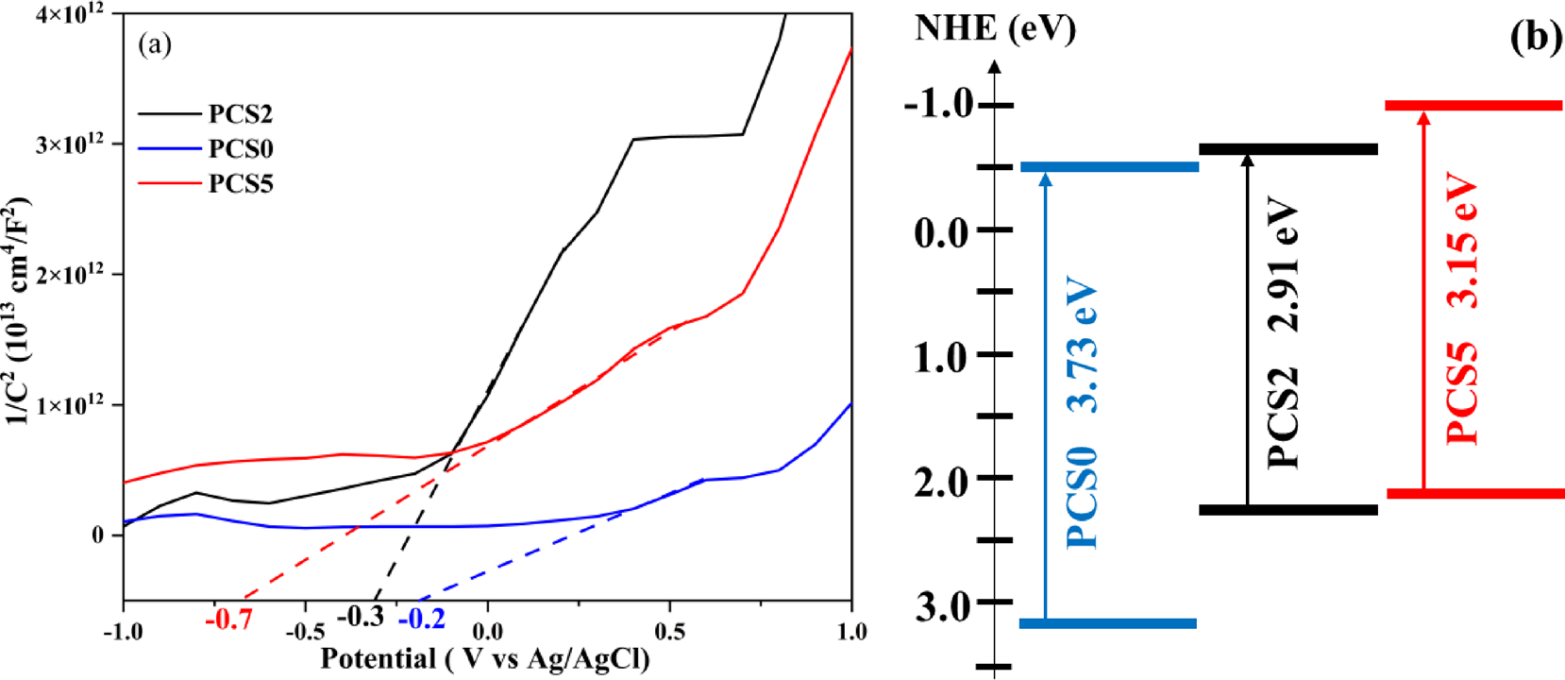
(**a**) Mott-Schottky plots of PCS0, PCS2, and PCS5 composites, and (**b**) band structure diagrams of PCS0, PCS2, and PCS5 composites.

Electrochemical property is considered another important factor that determines the performance of semiconductor photocatalysts. Mott-Schottky plots (Fig. 8a) were used to determine the density of charge carriers of the samples using Eq. (3):

17 $${N}_{d}=\left(\frac{2}{e\varepsilon {\varepsilon }_{0}}\right){\left[\frac{d\left({C}^{2}\right)}{dV}\right]}^{-1}$$

where e_0_, ε, and ε_0_ are the electron charge, the dielectric constant of the sample, and the permittivity of the vacuum, respectively; [d(C^2^)/dV]^−1^ is the slope of the Mott-Schottky plots. The linear part of the Mott-Schottky curve had a positive slope, revealing that all the as-prepared products were n-type semiconductors. The flat band potential in n-type semiconductors is 0.3 electron volts higher than the conduction band potential^75–77^. So, with the following equations, Table 3 has been completed:

**Table 3:** Electrochemical properties of PCS0, PCS2, and PCS5 composites obtained from Mott-Schottky analyses.

Composite	Flat band potential	Conduction band	Band gap	Valence band	Slope of the diagram	Dielectric constant (ε)	Charge carrier density
PCS0	− 0.2	− 0.5	3.73	3.43	2.2	5.14	2.74 × 10^29^
PCS2	− 0.3	− 0.6	2.91	2.51	5.1	6.9	2.04 × 10^29^
PCS5	− 0.7	− 1	3.15	2.35	4.12	6.11	2.30 × 10^29^

18 $${E}_{CB}={E}_{FB}-0.3$$

19 $${E}_{CB}={E}_{g}-{E}_{VB}$$

20 $${N}_{D}=\frac{2}{m{\varepsilon }_{r}{\varepsilon }_{0}e}$$

According to Eq. (17), e and ε_0_ are constant and equal to 1.602 × 10^−19^ C and 8.85 × 10^−12^ Fm^−1^, respectively. By using mott-Schottky and band gap data, the location of the conduction and valance band of composites is determined and shown in Fig. 8b. PCS0 has the highest charge carrier density at 2.74 × 10^29^, followed by PCS5 with at 2.30 × 10^29^ suggesting a greater number of carriers available for electron transfer but they coupled with a high bandgap, limiting their visible-light activity. Although PCS2 has the lowest density (2.04 × 10^29^), it has the lowest band gap, which can work in the visible-light spectrum. The narrower bandgap enables PCS2 to capture lower-energy photons efficiently, while its conduction band edge (0.6 eV) and valence band edge (2.51 eV) align well for visible-light-driven photocatalysis.

### Photocatalytic degradation of TC

**Fig. 9:**
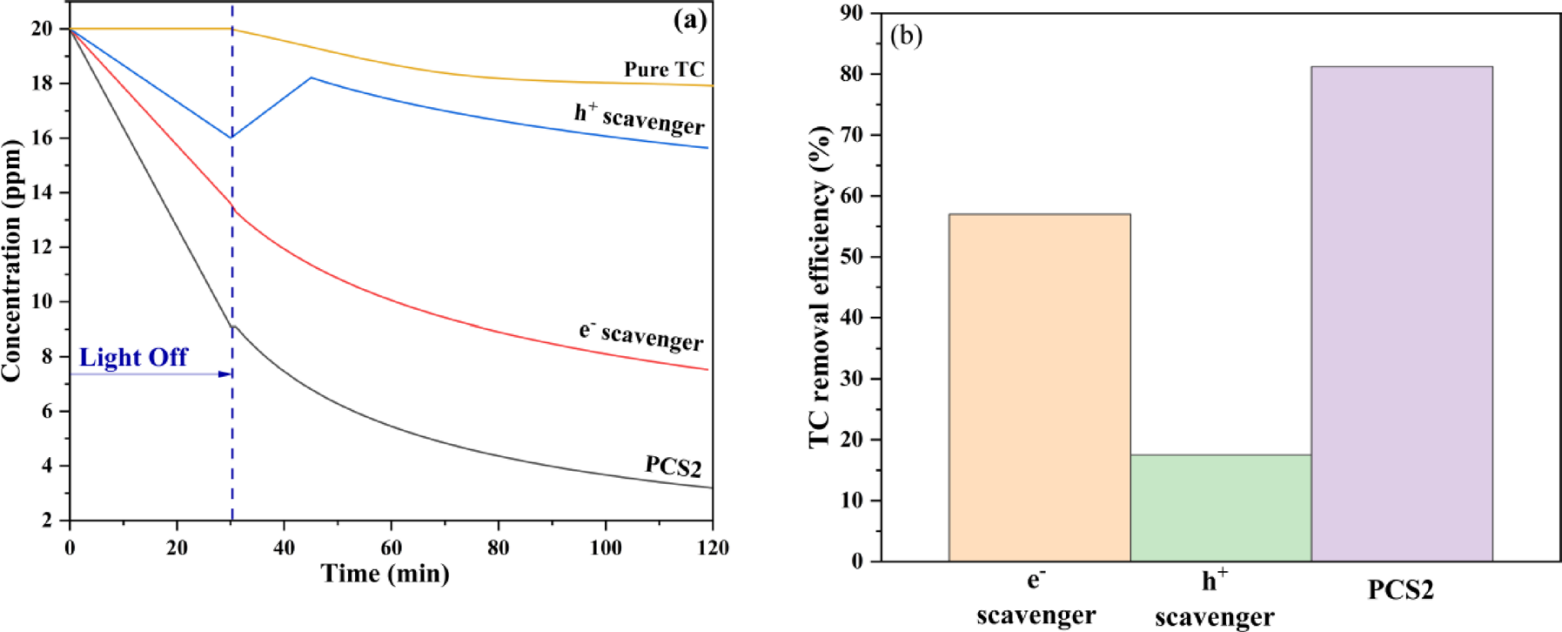
(**a**) Time-dependent photocatalytic degradation curves and (**b**) degradation efficiency of pure TC, PCS2 composite, hole, and electron scavengers.

**Fig. 10:**
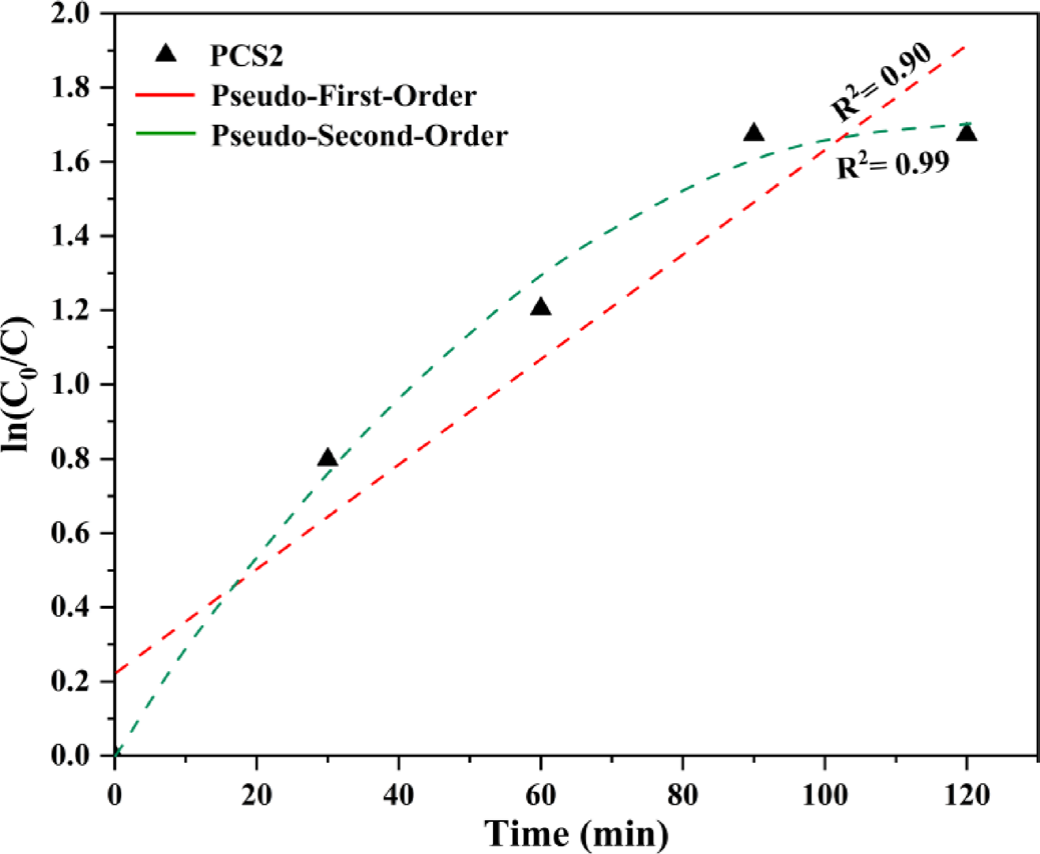
Time-dependent kinetics curve for the PCS2 composite, which shows pseudo-second-order kinetic plots.

The photodegradation of TC over the PCS2 nanocomposite was evaluated under visible light irradiation. The UV–visible spectrum of the TC solution (initial concentration 20 ppm, 100 ml) is shown in Fig. 9a. There is a slight TC degradation over the illumination of visible light without the presence of the nanocomposite. After adding the PCS2 nanocomposite (Fig. 9a, black line), the solution was stirred for 30 min in the dark to reach the adsorption equilibrium, which adsorbed about 55% of TC. The high amount of adsorption is related to the high specific surface area of the nanocomposite, which was proved by N_2_ adsorption–desorption analysis Table 1. Under dark conditions, porous carbon primarily contributes to tetracycline adsorption within the composite. Its high surface area likely boosts the composite’s ability to capture organic molecules like tetracycline through mechanisms such as physical adsorption and π−π interactions^78^. Additionally, sulfur in ZnS or MoS_2_ can introduce functional groups and active sites that improve TC adsorption. These sulfur-based sites can form hydrogen bonds and increase the affinity between the composite surface and TC molecules, particularly with functional groups that interact effectively with sulfur^79^. The reaction was done, and after 90 min of illumination, the degradation reached 81.2%. This amount shows the effect of the nanocomposite, which consists of ZnS/MoS_2_ semiconductors, on the photocatalytic degradation of TC. Both MoS_2_ and TC molecules have the same bandgap of 1.9 eV, but TC cannot be degraded greatly by itself. Additionally, modification with sulfur mainly reduces the bandgap of the semiconductor. By using this composite and employing bandgap engineering, this level of degradation has been achieved. The primary active species responsible for degradation was identified by introducing h^+^ and e^−^ scavengers, illustrated in Fig. 9a. Holes were found to be the dominant reactive species, as the addition of a hole scavenger reduced degradation efficiency to 18%, highlighting their critical role in the degradation reaction. Conversely, the addition of an electron scavenger still enabled 57% degradation, indicating that while electrons contribute to the process, holes remain the principal active species. Degradation efficiency in the presence of PCS2, electron, and hole scavengers is shown in Fig. 9b. The kinetic analysis, depicted in Fig. 10, provides insight into the degradation efficiency of the PCS2 composite. The corresponding regression correlation coefficient (R^2^) values from the kinetic investigation demonstrated that kinetics followed pseudo-second-order. According to Fig. 10, R^2^ values were 0.9 and 0.99 for the pseudo-first and pseudo-second-order reaction of TC, respectively. The rate constant for the PCS2 composite without any scavengers was 0.00361 min^−1^, indicating a robust reaction rate.

**Fig. 11:**
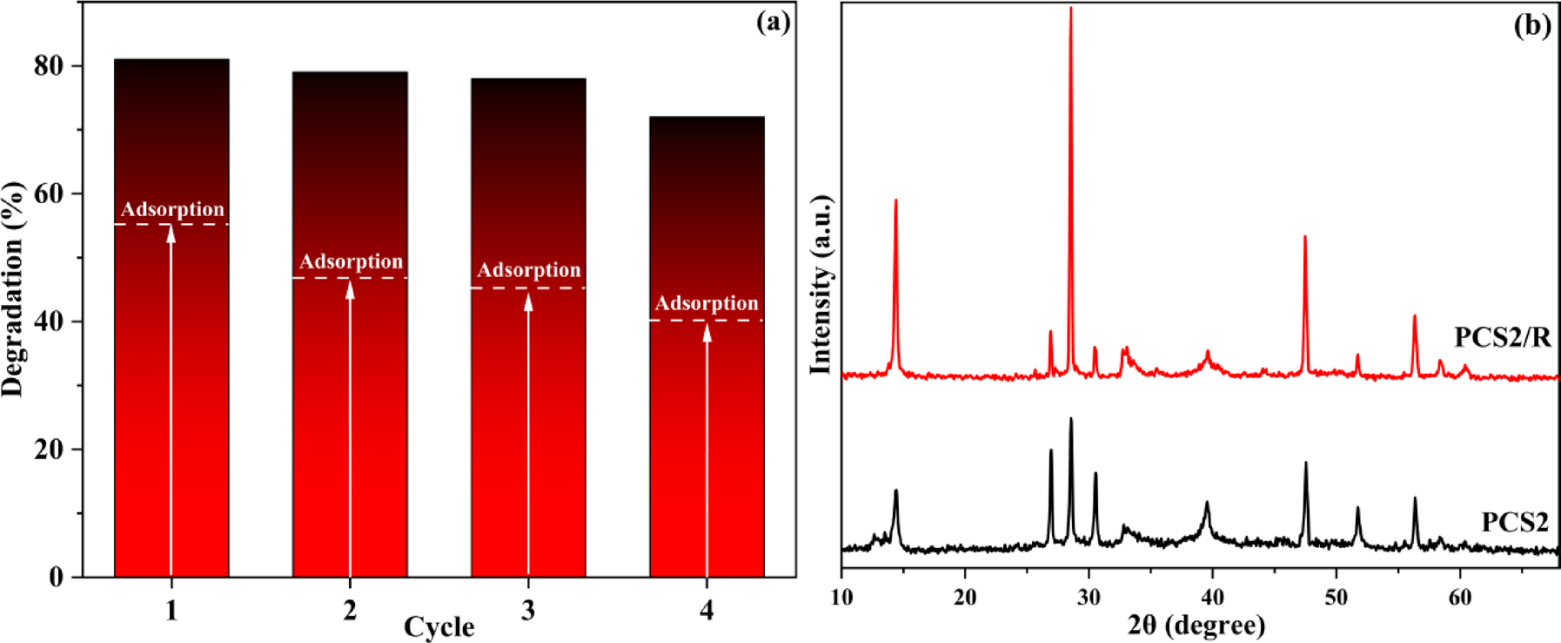
(**a**) Reusability performance of PCS2 composite in four consecutive photocatalytic cycles for tetracycline degradation under visible light irradiation (TC = 20 ppm), and (**b**) XRD patterns of PCS2 before and after four photocatalytic cycles, confirming the structural stability of the composite with only slight variations in peak intensity.

It is well established that one of the most essential factors influencing a catalyst’s practical utility is its stability and reusability. Four cycle tests were conducted to verify the reusability of PCS2. The final result is displayed in Fig. 11a. Following four cycle studies, the TC adsorption was 55%, 48.3%, 45%, and 40.4%, and the total TC degradation efficiency was 81.2%, 79.1%, 78.1%, and 72.3%, respectively. It demonstrated that there was no significant drop in PCS2’s catalytic activity. After four photocatalytic cycles, no significant changes in PCS2 structure are indicated by the XRD results (Fig. 11b), which show that the composite has high structural stability during the photocatalytic process, and only small differences in the intensity of some peaks have been formed. These modifications are due to some reasons, for example, reaction products or impurities may be generated on the surface of the composite during photocatalytic reaction and alter the XRD pattern. Also, aggregation or redistribution of active particles in cycles may alter the XRD pattern. This result indicates that the nanocomposites maintained their structural and chemical stability under light irradiation, showing strong resistance to photodegradation. By preserving their integrity, they effectively avoided the risk of photocorrosion typically caused by extended light exposure^80–83^.

## Possible adsorption/photodegradation mechanism

**Fig. 12:**
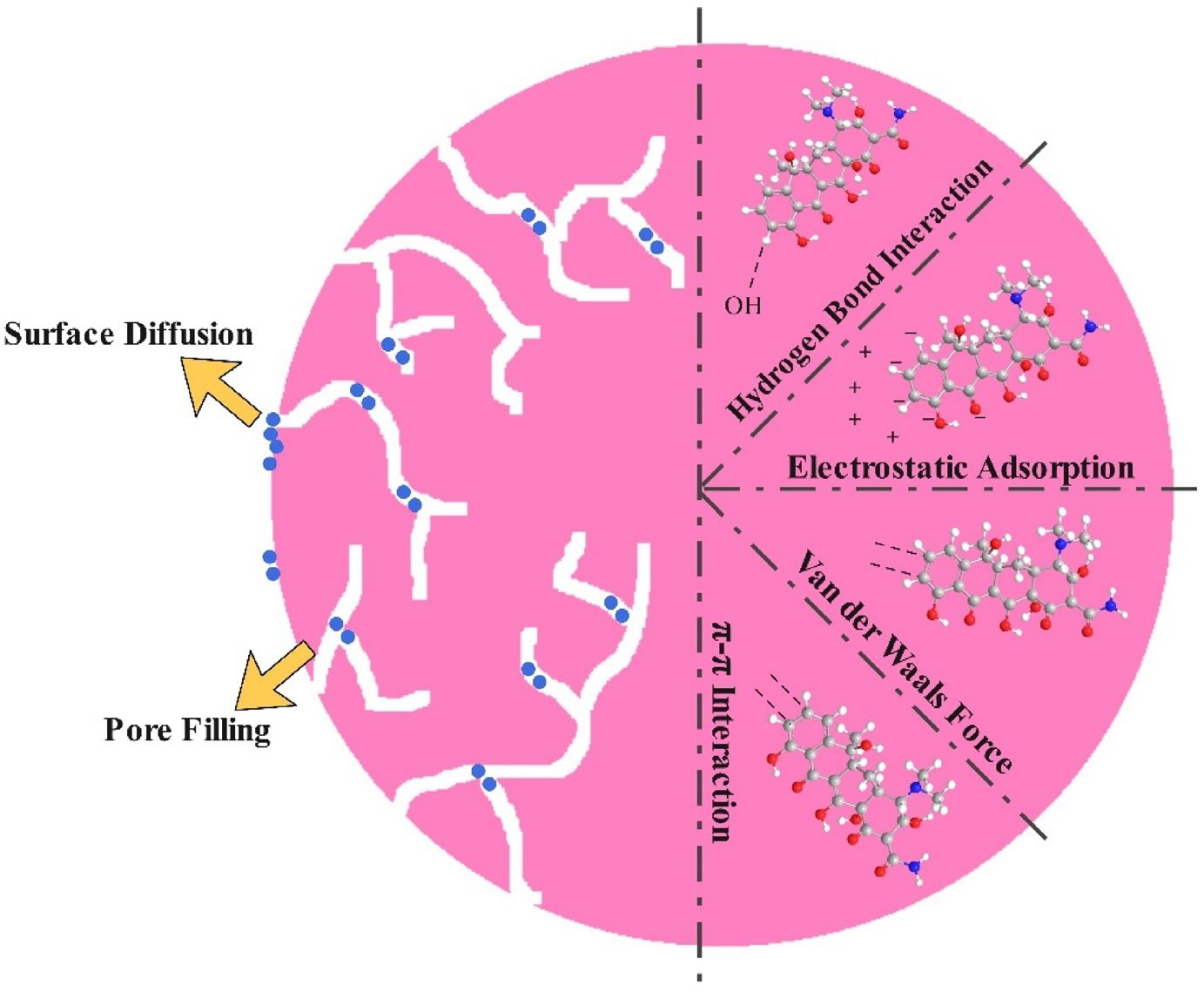
Schematic illustration of the proposed adsorption mechanism of tetracycline onto porous carbon, involving surface adsorption and subsequent diffusion of TC molecules into the internal pore structures.

The literature widely reports that the adsorption of tetracycline (TC) onto carbon-based composites occurs through H-bonding, pore filling, π−π interaction, and electron attraction ^84^. Larger surface areas (216.83 m^2^ g^−1^) and greater pore volumes (0.16 cm^3^ g^−1^) significantly improve adsorption capacity through the pore-filling effect, which is particularly important given the relatively large molecular size of TC. Additionally, a lower polarity index—indicated by reduced O/C and (O + N)/C atomic ratios—benefits adsorption since higher polarity leads to the formation of water clusters via hydrogen bonding, creating a barrier that limits TC’s access to the adsorbent surface. Furthermore, a lower H/C ratio, reflecting higher aromaticity, provides more graphitic (π-donor) sites that can engage in π–π electron donor–acceptor (EDA) interactions with TC molecules, which act as π-acceptors because of their ketone functional groups (Fig. 12)^85^.

From the aspect of degradation, when a semiconductor molecule absorbs electrons with energy equal to or higher than its band gap, the valence band electrons can be excited and transferred into the conduction band, and thus, charge carriers are produced. Electron–hole recombination after their separation should be prevented as much as possible to have a photocatalyzed reaction. Generally, under light irradiation, the photogenerated electrons in ZnS are excited from the valence band (VB) to the conduction band (CB), leaving behind holes in the VB. Since the conduction band of MoS_2_ lies at a more negative potential than that of ZnS, these photoexcited electrons transfer from the CB of ZnS to the CB of MoS_2_, while the photogenerated holes move from the VB of ZnS to the VB of MoS_2_. This efficient charge separation process inhibits the rapid recombination of electron–hole pairs, thereby enhancing the overall photocatalytic performance. The separated charge carriers then participate in surface redox reactions. The transferred electrons on MoS_2_ react with adsorbed oxygen molecules to generate reactive oxygen species (ROS), such as superoxide radicals, while the holes oxidize water molecules or hydroxyl ions to produce hydroxyl radicals. These reactive species subsequently degrade adsorbed TC molecules into non-toxic products like CO_2_ and H_2_O^86–89^. Electron–hole recombination occurs unless oxygen is available to remove electrons to form the superoxide radical (^·^O_2_^−^) (Eq. 21), which then reacts with H^+^ to form the hydroperoxyl radical (OOH^·^) (Eq. 22). Then H_2_O_2_ is formed according to Eq. (23).

In the degradation of organic pollutants, the hydroxyl radical is produced from the reduction of H_2_O_2_ and the oxidation of adsorbed water or hydroxide (Eqs. 24–26) (OH^·^ is the primary oxidizer). Positive holes can oxidize organic pollutants directly (Eq. 27). On the other hand, OH^·^ attacks organic compounds (Eq. 28), leading to different reactions depending on the nature of the compounds. The resulting intermediates react more with OH^·^ than final degradation products, such as CO_2_ and H_2_O. In the photocatalytic decomposition of pollutants, when oxygen reduction and pollutant oxidation do not proceed simultaneously, electron accumulation occurs in the conduction band and thus increases the recombination rate of e^−^ and h^+^. Therefore, simultaneous oxygen reduction and pollutant oxidation reactions are very important. The chain reactions are described in full below.

21 $${\text{O}}_{{2}} + {\text{e}}^{ - } \to \,^{\cdot}{\text{O}}_{{2}}^{ - }$$

22 $$\,^{\cdot} {\text{O}}_{2}^{ - } + {\text{H}} + \leftrightarrow \,^{\cdot} {\text{OOH}}$$

23 $${2} \,^{\cdot} {\text{OOH}} \to {\text{H}}_{{2}} {\text{O}}_{{2}} + {\text{O}}_{{2}}$$

24 $${\text{H}}_{{2}} {\text{O}}_{{2}} + {\text{ e}}^{ - } \to \,^{\cdot} {\text{OH}} + {\text{OH}}^{ - }$$

25 $${\text{H}}_{{2}} {\text{O }} + {\text{ h}}^{ + } \to \,^{\cdot} {\text{OH}} + {\text{ H}}^{ + }$$

26 $${\text{OH}}^{ - } + {\text{ h}}^{ + } \to \,^{\cdot} {\text{OH}}$$

27 $${\text{R}}^{ \cdot } + {\text{ h}}^{ + } \to {\text{R}}^{ + }$$

28 $${\text{Organic }} + {\text{ radicals }}( \,^{\cdot} {\text{OOH}}, \,^{\cdot} {\text{OH}}) \to {\text{Degradation product}} \to {\text{CO}}_{{2}} + {\text{ H}}_{{2}} {\text{O}}$$

**Fig. 13:**
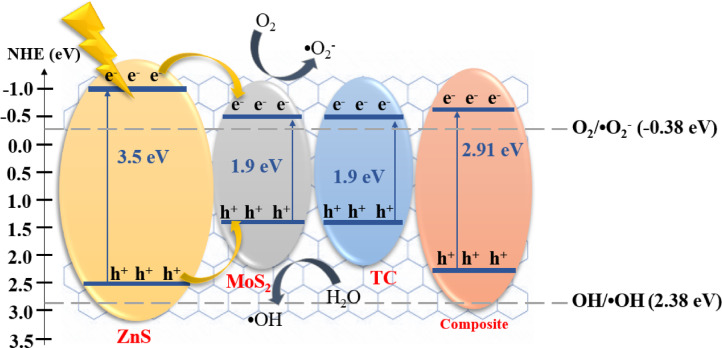
Schematic of the reaction mechanism for TC degradation.

As seen in Fig. 13, both oxidation and reduction reactions can take place on the excited semiconductor photocatalyst surface.

## Comparison of photocatalysts for TC degradation

Table 4 compares the photocatalytic performance of PCS2 with other photocatalysts reported in the literature for TC degradation. It includes information on exposure time, specific surface area, degradation efficiency, catalyst dosage, and initial tetracycline concentration. PCS2 has achieved an impressive 81% tetracycline degradation efficiency without any optimization of operational parameters, highlighting its inherent photocatalytic potential.

**Table 4:** The photocatalytic performance of PCS2 to TC degradation compared to previous works.

Composites	Exposure time	Specific surface area (m^2^ g^−1^)	Degradation (%)	Catalyst dosage (mg L^−1^)	Tetracycline dosage (mg L^−1^)	References
Corchorus olitorius-derived Biochar/Bi_12_O_17_Cl_2_	360 min	277.6	85.8	100	167	^90^
Fe_3_O_4_/g-C_3_N_4_/MoO_3_ (30%)	120 min	72.6	94	20	40	^91^
1.0 wt% Ag/Bi_3_O_4_Cl	120 min	–	94.2	500	10	^92^
ZnO/NiFe_2_O_4_/Co_3_O_4_	20 min	–	98	20	30	^93^
Cr_2_O_3_/ZrO_2_	120 min	38.4	97.1	100	50	^94^
GdFeO_3_/WS_2_	90 min	178.9	96.2	40	10	^95^
MnFe_2_O_4_/BGA (boron-doped graphene aerogel)	60 min	142.4	92.15	200	20	^96^
TiO_2_@SCN	60 min	–	98.1	100	10	^97^
ZnS/MoS_2_-Decorated Porous Carbon	120 min	216.83	81	100	20	This work

## Conclusion

In this research, we investigated the synthesis of a ZnS/MoS_2_ nanocomposite on a porous carbon substrate through a one-step CVD-inspired green pyrolysis method, utilizing melamine and zinc nitrate precursors with varying sulfur content. The findings demonstrated that the amount of sulfur significantly influenced the synthesized composite’s optical and electrochemical properties. While the addition of sulfur increased the specific surface area, it was observed that exceeding the optimal amount led to a reduction in surface area. The optimal composite, PCS2 (synthesized with 2 g of sulfur), exhibited a remarkable band gap of 2.91 eV and a high specific surface area of 216.83 m^2^ g^−1^, positioning it as the most effective material among the tested samples. The PCS2 sample exhibits mesoporosity and microporosity volumes of 0.124 cm^3^ g^−1^ and 0.036 cm^3^ g^−1^, respectively. FESEM analysis reveals that ZnS and MoS_2_ are well-integrated, forming a heterojunction nanocomposite structure. Also, based on FESEM micrographs, MoS_2_ displays its characteristic 2D sheet-like morphology due to its hexagonal crystal structure. HRTEM images confirm the lattice spacings of the composite components: 0.33 nm for porous carbon, 0.16 nm for ZnS, and 0.27 nm for MoS_2_. These distinct lattice spacings validate the formation of a well-integrated heterojunction structure, with ZnS and MoS_2_ efficiently incorporated into the carbon matrix. PCS2 effectively mineralized tetracycline (TC) into smaller intermediates or directly into CO_2_ and H_2_O under visible light, achieving an impressive total removal efficiency of 81% for tetracycline at a concentration of 20 ppm, with approximately 55% of this removal attributed to the adsorption process. The introduction of scavengers during the degradation experiments revealed that holes were the primary active species responsible for the photocatalytic activity. Overall, the synthesized ZnS/MoS_2_ heterojunction composite demonstrates significant potential for wastewater treatment applications, providing an effective strategy for addressing the environmental challenges posed by residual antibiotics. However, there are still several areas that require further exploration. Although the optimal sulfur content for the composite herein has been identified, it would be valuable to explore deeper into understanding how sulfur specifically interacts with the other components. Moreover, the long-term stability and reusability of the composite under real-world degradation conditions remain important factors that need to be explored, as these are crucial for practical wastewater treatment applications. Lastly, combining this composite with other technologies like membrane filtration could significantly boost its performance and broaden its potential use in environmental cleanup.

## Data Availability

All data presented in this study are included in this published article. Furthermore, additional data supporting the plots and other findings presented in this paper are available from the corresponding author upon reasonable request. For inquiries regarding the data, please contact the following email: 777Zahraamiri@gmail.com m_safarzadeh@nt.iust.ac.ir.
